# Health-related quality of life in patients with chronic hepatitis C treated with sofosbuvir-based treatment at 1-year post-sustained virological response

**DOI:** 10.1007/s11136-021-02874-6

**Published:** 2021-05-19

**Authors:** Akio Miyasaka, Yuichi Yoshida, Akiko Suzuki, Yasuhiro Takikawa

**Affiliations:** Division of Hepatology, Department of Internal Medicine, Iwate Medical University School of Medicine, 2-1-1 Idaidori, Yahaba-cho, Shiwa-gun, Iwate, 028-3695 Japan

**Keywords:** HCV, Sofosbuvir, Ribavirin, Ledipasvir, HRQoL, SF-8

## Abstract

**Purpose:**

Long-term effects on patient health-related quality of life (HRQoL) after direct-acting antiviral (DAA) treatment for hepatitis C virus (HCV) are unknown. We assessed the impact of DAA-mediated HCV clearance on HRQoL from DAA initiation to 1 year after confirmed sustained virological response at 24 weeks post-treatment (SVR24).

**Methods:**

HRQoL was evaluated using the eight-item Short Form Health Survey (SF-8). Chronic HCV-infected patients were treated for 12 weeks with sofosbuvir-based DAAs. SF-8 was administered at baseline, treatment cessation, SVR24, and 1-year post-SVR24.

**Results:**

A total of 109 chronic HCV-infected patients were enrolled. The average SF-8 scores were higher than the Japanese national standard values for bodily pain (BP) and mental health at baseline and for general health at 1-year post-SVR24. None of the SF-8 scores differed significantly between baseline and 1-year post-SVR24. Regarding age, sex, liver status, and treatment regimen, the SF-8 scores at 1-year post-SVR24 were affected by only age; individuals aged < 65 years had significantly higher physical component score (PCS), physical functioning, role physical, and BP scores than older individuals. In the multivariable analysis, only age of ≥ 65 years was significantly associated with influencing PCS at 1-year post-SVR24. However, no significant factors were identified for mental component score.

**Conclusion:**

Upon long-term assessment, although more factors trended higher than national standard values at 1-year post-SVR24 than at baseline, there were no significant changes within factors. As PCS tended to be associated with age, patients aged ≥ 65 years should be carefully monitored for PCS.

## Plain English summary

Interferon (IFN)-free direct-acting antiviral (DAA) treatment for hepatitis C virus (HCV) infection leads to fewer side effects and higher treatment efficacy compared with IFN treatment. There have been many reports regarding health-related quality of life for DAA treatment up until viral clearance in the blood, sustained at 24 weeks after treatment completion, but the effects of DAA treatment on long-term quality of life remain unknown. In this study, we assess the impact of DAA-mediated HCV clearance on health-related quality of life from treatment initiation to 1 year after confirmed viral clearance using an eight-point Short Form Health Survey. We found that more categories were higher than national standard values at 1-year post-clearance than at baseline, but there were no significant changes within categories. Our findings highlight that the mental component, including vitality, social functioning, role emotional, and mental health, should be carefully monitored, while poor scores in the physical categories including physical functioning, role physical, bodily pain, and general health, tended to be associated with age, and so elderly patients aged ≥ 65 years should be carefully monitored for these aspects.

## Introduction

Hepatitis C virus (HCV) is a leading cause of hepatic cirrhosis, hepatocellular carcinoma, and end-stage liver disease [1, 2]. The introduction of interferon (IFN)-free direct-acting antiviral (DAA) regimens has changed the treatment of HCV infection. In 2014, the combined use of asunaprevir (an NS3/4A protease inhibitor) and daclatasvir (an NS5A inhibitor) became the first approved IFN-free treatment for genotype (GT) 1 HCV infection in Japan. Thereafter, sofosbuvir (SOF; an NS5B polymerase inhibitor) and ribavirin (RBV) [3] were indicated for GT2 HCV infection, and ledipasvir (LDV; an NS5A inhibitor)/SOF [4] were approved for use in patients with GT1 HCV infection. Subsequently, DAAs for HCV treatment were released one after another. All these treatments show high efficacy and safety, with > 90% patients attaining a sustained virologic response (SVR), defined as the elimination of HCV at a particular timepoint after treatment completion, such as 24 weeks (SVR24).

Health-related quality of life (HRQoL) has become an increasingly important metric for assessing the impact of a chronic disease or status on affected patients after therapeutic interventions. IFN-based treatments lead to a decrease in HRQoL owing to the incidence of adverse reactions [5–7]; conversely, it has been reported that DAA treatment does not decrease HRQoL during treatment [8]. Other reports show that HRQoL was improved at SVR12–24 [9, 10], and one report indicates that the degree of improvement in HRQoL at SVR12 differs depending on the treatment regimen [11]. However, these reports assessed HRQoL only until SVR12–24. Although eliminating HCV via DAA treatment may improve patient HRQoL past this point, the long-term effect of DAA treatment for HCV on patient HRQoL is unknown. Therefore, this study aimed to assess the long-term impact of HCV clearance following SOF-based treatment on HRQoL at 1-year post-SVR24.

## Methods

### Patients and procedures

Patients aged over 20 years old with chronic hepatitis (CH) and compensatory liver cirrhosis (LC) who were treated with LDV/SOF (Gilead Sciences, Tokyo, Japan) for GT 1 or SOF/RBV (MDS, Tokyo, Japan) for GT 2 were recruited for our longitudinal study. All enrolled patients were treated at Iwate Medical University in Iwate Prefecture, Japan, during the period from October 2015 to November 2017.

All patients who were given LDV/SOF or SOF/RBV orally for 12 weeks were treatment naïve. HRQoL was measured at baseline, end of treatment (EOT), 24 weeks after EOT (SVR24), and 76 weeks after EOT (1-year post-SVR24).

The required sample size (n) was calculated using the statistical software G*Power (Heinrich Heine University, Düsseldorf, Germany) [12]. The results of these calculations indicate that, for an effect size (η) of 0.25 (indicative of a moderate-sized effect on HRQoL), α-error probability of 0.05, and power of 80%, the required sample size is 36. Therefore, we determined that this study would require at least 36 samples.

Serum HCV RNA levels were measured using a COBA TaqMan HCV quantitation assay (Roche Diagnostics, Tokyo, Japan). HCV RNA was examined every 4 weeks from treatment initiation until 36 weeks post-initiation. SVR24 was defined as undetectable HCV RNA at 24 weeks after treatment completion. Patients with SVR24 were analyzed to investigate factors associated with changes in HRQoL.

The study was conducted in accordance with the Declaration of Helsinki and approved by the Ethics Committee of Iwate Medical University (approval numbers H27-135 and H27-147). All patients provided a signed written informed consent form before the start of the study.

### Assessment of parameters from baseline to 1-year post-SVR24

Laboratory data and anthropometric measurements were obtained at baseline, EOT, SVR24, and 1-year post-SVR24. Body mass index was calculated from the patient weight in kilograms divided by the square of patient height in meters. HCV genotype was determined before treatment. Laboratory examinations included assessments of white blood cell and platelet counts and levels of hemoglobin, albumin, alanine aminotransferase (ALT), aspartate aminotransferase (AST), and alpha-fetoprotein. The fibrosis-4 (Fib-4) index was calculated by applying the following formula: (AST × Age)/(Platelet counts × √ALT). The presence of LC was evaluated at screening based on different combinations of liver biopsy findings, Fib-4 index, serum markers of fibrosis, transient elastography, liver imaging examination, and clinical state.

### HRQoL assessment

There are several patient-reported methods of assessing outcomes from chronic disease. We used a Japanese-validated version of the eight-item Short Form Health Survey (SF-8) to evaluate the HRQoL of enrolled patients [13]. The SF-8 has been extensively validated and widely used for obtaining a self-reported assessment of HRQoL for patients in various settings [14]. In addition, the use of SF-8 here allowed the scores from this work to be compared with the Japanese national standard value for SF-8.

The survey was conducted at each of the four instances when the enrolled patients visited our hospital. Each patient completed the SF-8 questionnaire by themselves. The same clinician consistently collected and managed the data throughout the whole study period. The SF-8 questionnaire assessed eight health factors: physical functioning (PF), role physical (RP), bodily pain (BP), general health (GH), vitality (VT), social functioning (SF), role emotional (RE), and mental health (MH). The physical component score (PCS) and mental component score (MCS) were each calculated from the sum of the scores of the corresponding domains: PF, RP, BP, and GH for the PCS, and VT, SF, RE, and MH for the MCS. The Japanese national standard values for the PCS and MCS are each 50 points, with standard deviations of 10 points. Higher scores are considered to indicate a better HRQoL. The treating clinician or an appropriately trained staff member evaluated patients based on their responses to the SF-8 questionnaire. Changes in single SF-8 factors between baseline and 1-year post-SVR24 were considered to reflect inter-individual change, whereas such differences in two groups were considered to reflect intra-individual change. Thus, the change in each SF-8 factor from baseline to 1-year post-SV24 was evaluated in collective group scoring, and all other assessments were performed on the 42 participants for whom complete data were available.

### Statistical analysis

SPSS software (IBM Japan, Tokyo, Japan) was used for statistical analyses. Continuous variables are described as the mean ± standard deviation (SD) or median (range), and a Student’s *t*-test was employed for tests of significance. Regarding the average changes for each SF-8 factor, data were analyzed by conducting a repeated measure ANOVA, followed by a Bonferroni’s multiple comparison post hoc test, and the effect size (η^2^) was simultaneously calculated. Herein, Bonferroni’s correction was applied, in which *α* = 0.05/3 = 0.017 was set as the level of significance. For differences in each SF-8 factor from baseline to each timepoint, statistical significance was examined using a paired *t*-test, and the effect size (*d*) was also calculated. Intergroup comparisons were performed using Student’s *t*-test, and the effect size (*d*) was analyzed. Categorical variables are described as ratios and were analyzed using a Fisher’s exact test. The following potential and clinically important factors at 1-year post-SVR24 were categorized into two groups: Age (≥ 65 years old or < 65 years old), Sex (male or female), Liver state (CH or LC), and Treatment regimen (LDV/SOF or SOF/RBV). Regarding whether a given factor influenced PSC or MCS, improvement or worsening was defined by whether the average score at 1-year post-SVR24 was higher or lower, respectively, than the average score at baseline. After univariate analysis, multivariable logistic-regression models were applied to estimate the factors associated with influencing SF-8 parameters (PCS and MCS), including sociodemographic variables (i.e., age and sex) and clinical variables (i.e., liver status and treatment regimen), which excluded potential confounding factors associated with liver fibrosis. This model is reported as the odds ratio, 95% confidence interval, and *p*-value. Values of *p* < 0.05 were considered statistically significant. Graphs were constructed using GraphPad Prism (GraphPad Software Inc., San Diego, CA, USA).

## Results

### Patient characteristics

This study included 109 participants at baseline; 105 remained at EOT, 95 at SVR24, and 42 at 1-year post-SVR24. The main reasons for participant drop-out were failure to complete the SF-8 questionnaire at EOT (drop-out rate of 2%) or SVR24 (drop-out rate of 13%) and loss to follow-up at 1-year post-SVR24 (drop-out rate of 62%). The baseline clinical and demographic characteristics of the participants are shown in Table 1. A total of 109 patients were initially enrolled in this study. The median age of this cohort was 67 years old, with 56% of patients aged ≥ 65 years old; 41% were males, 28% were cirrhotic at baseline. The 80 patients with GT 1 were treated with SOF/LDV, and the 29 patients with GT 2 were treated with SOF/RBV.

**Table 1:** Clinical and demographic characteristics from baseline to 1-year post-SVR24

	Baseline (*n* = 109)	Baseline Completed follow-up (*n* = 42)	Baseline Failed to complete follow-up (*n* = 67)	EOT (*n* = 105)	SVR24 (*n* = 95)	1-year post-SVR24 (*n* = 42)
Age (years)#	67 (21–86)	68 (37–82)	66 (21–86)	67 (21–86)	66 (22–84)	67 (39–85)
Sex (male/female)	45/64	15/27	30/37	42/63	37/58	15/27
Body mass index (kg/m^2^)#	23.3 (16.3–36.6)	23.0 (17.0–36.6)	23.6 (16.3–34.6)	22.6 (16.1–35.8)	22.9 (17.1–36.7)	22.4 (16.2–33.0)
White blood cells (/µL)#	4610 (1010–8230)	4310 (7380–2840)	4640 (1010–8230)	4555 (1270–14,260)	4990 (1740–11,900)	4690 (2390–16,700)
Hemoglobin (g/dL)#	13.2 (9.7–17.7)	13.4 (9.7–17.0)	13.1 (10.1–17.7)	12.6 (9.6–17.4)	13.4 (9.1–43.7)	13.5 (10.5–16.7)
Platelets (× 10^4^/µL)#	15.0 (4.5–34.1)	15.1 (4.5–28.6)	14.6 (3.5–34.1)	18.0 (4.4–41.9)	17.5 (4.4–40.2)	18.1 (5.5–29.0)
Albumin (g/dL)#	4.0 (3.0–5.0)	4.1 (3.2–4.7)	4.0 (3.0–5.0)	4.2 (3.3–4.9)	4.2 (3.1–5.0)	4.3 (3.8–4.9)
Aspirates aminotransferase (U/L)#	37 (15–160)	38 (17–1156)	36 (15–160)	22 (11–211)	24 (12–116)	23 (12–83)
Alanine aminotransferase (U/L)#	36 (11–237)	40 (11–196)	35 (12–237)	16 (7–77)	18 (6–73)	18 (8–74)
Alpha-fetoprotein (ng/mL)#	3.8 (1.2–125.5)	3.9 (1.4–67.6)	3.6 (1.2–125.5)	3.0 (1.0–39.6)	4.1 (0.9–54.7)	3.2 (1.8–7.3)
HCV RNA (Log IU/mL)#	6.1 (3.2–7.3)	6.1 (3.6–7.3)	6.1 (3.2–7.1)	0	0	0
HCV genotype (1/2)	80/29	36/6*	44/23*	79/26	73/22	6/36
Chronic hepatitis/Liver cirrhosis	78/31	30/12	48/19	77/28	67/28	30/12
Fib-4 index	2.74 (0.32–19.66)	2.76 (0.68–12.6)	2.70 (0.33–19.66)	2.22 (0.32–20.0)	2.91 (0.37–19.52)	2.40 (0.61–10.20)
Treatment history of HCV (Yes/No)	30/79	12/30	18/49	30/75	24/71	12/30
Treatment history of HCC (Yes/No)	14/95	3/39	11/56	13/92	13/82	3/39

*HCV* hepatitis C virus, *HCC* hepatocellular carcinoma, *EOT* end of treatment, *SVR24* sustained virological response at 24 weeks after treatment completion

*p < 0.05 in a Fisher’s exact test between patients who completed follow-up and those who failed to complete follow-up

^#^Data are expressed as the median (range)

The clinical and demographic characteristics of the participants at EOT, SVR24, and 1-year post-SVR24 are also shown in Table 1. Among the included patients, 27% (28/105) at EOT, 30% (28/95) at SVR24, and 29% (12/42) at 1-year post-SVR24, respectively, had cirrhosis. At the end of the study, we had obtained data from all four timepoints, (baseline, EOT, SVR24, and 1-year post-SVR24) for 42 patients. Of those 42 patients, the median age was 67 years old, with 55% of patients aged ≥ 65 years old; 36% were males. None of these 42 patients experienced hepatocellular carcinoma.

### Changes in HRQoL

The mean SF-8 scores for each timepoint from baseline to 1-year post-SVR24 are shown in Table 2. In comparison with the Japanese national standard values, the average SF-8 scores at baseline were 0.01 to 2.88 points lower for PF, RP, GH, SF, RE, PCS, and MCS, whereas they were 0.16 to 1.81 points higher for BP, VT, and MH. These differences in the BP, MH, PF, RP, GH, SF, and RE scores and the PCS were statistically significant.

**Table 2:** Mean SF-8 factor scores from baseline to 1-year post-SVR24

	National standard value (*N* = 2284)	Baseline (*n* = 109)	EOT (*n* = 105)	SVR24 (*n* = 95)	1-year post-SVR24 (*n* = 42)	Effect size (η^2^)
PF	49.84 ± 6.81	46.96 ± 9.10^♯^	48.45 ± 5.90^♯^	47.60 ± 7.45^♯^	46.95 ± 9.20^♯^	0.04
RP	50.07 ± 6.58	48.30 ± 7.57^♯^	49.57 ± 5.80	48.07 ± 7.35^♯^	46.40 ± 9.54^♯^	0.07
BP	50.06 ± 8.55	51.87 ± 9.52^♯^	53.37 ± 8.00^♯^	51.48 ± 8.86^♯^	50.74 ± 8.71	0.18
GH	49.96 ± 7.29	48.77 ± 7.02^♯^	50.83 ± 6.97^♯^	52.00 ± 6.15*	51.64 ± 7.41^♯^	0.09
VT	50.09 ± 6.83	50.25 ± 6.15	50.55 ± 5.62	50.18 ± 6.48	50.73 ± 7.26	0.01
SF	50.00 ± 7.56	48.34 ± 8.59^♯^	49.08 ± 7.60^♯^	48.95 ± 7.54^♯^	48.27 ± 8.61^♯^	0.01
RE	49.94 ± 6.34	49.28 ± 5.34^♯^	50.56 ± 4.64^♯^	49.65 ± 5.59	48.69 ± 7.57^♯^	0.03
MH	49.70 ± 7.06	50.37 ± 6.32^♯^	52.10 ± 4.74^♯^	51.44 ± 5.75^♯^	50.45 ± 6.54	0.07
PCS	48.00 ± 7.24	47.31 ± 7.94^♯^	48.70 ± 6.24^♯^	47.76 ± 6.65	46.96 ± 8.31^♯^	0.07
MCS	49.44 ± 6.78	49.43 ± 6.88	50.46 ± 5.43^♯^	50.15 ± 5.72^♯^	49.72 ± 6.29	0.03

Data are expressed as the mean ± SD

Effect size (η^2^) was elevated from baseline to 1-year post-WVR24

*EOT* end of treatment, *SVR24* sustained virological response at 24 weeks after treatment completion, *PF* physical functioning, *RP* role physical, *BP* bodily pain, *GH* general health, *VT* vitality, *SF* social functioning, *RE* role emotional, *MH* mental health, *PCS* physical component score, *MCS* mental component score

*p = 0.0002 in repeated measured ANOVA when compared with the baseline level

^♯^*p* < 0.05 in Student’s *t*-test when compared with the National standard value

**Fig. 1:**
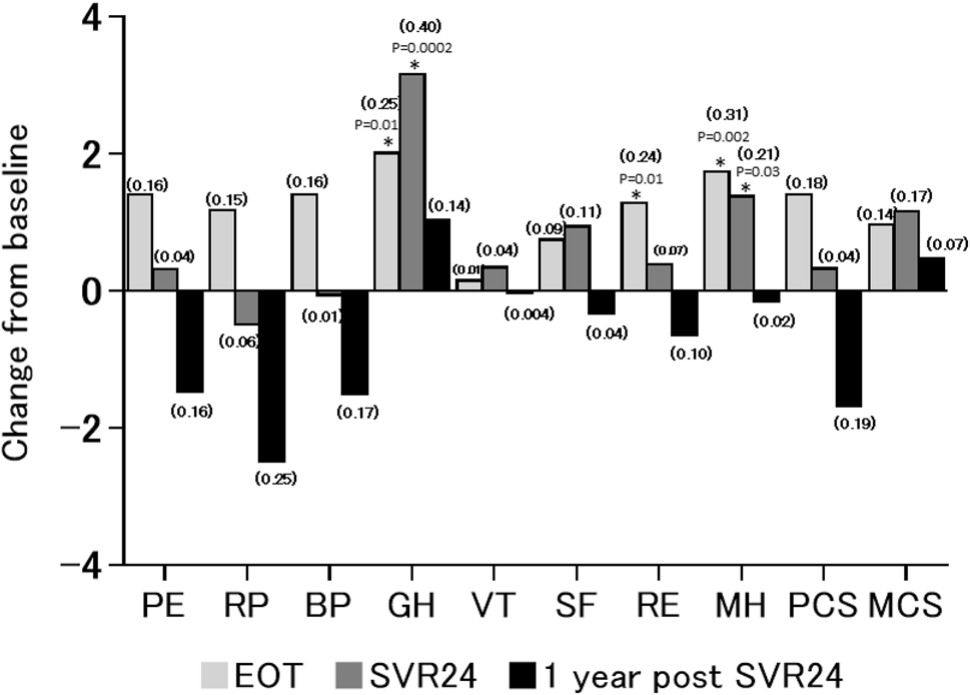
Differences in SF-8 scores between baseline and EOT, SVR24, or 1-year post-SVR24. Data are expressed as the mean of the absolute differences between baseline and the indicated timepoint. *p < 0.05 in a paired *t*-test when compared with the baseline level. The effect sizes are expressed in parentheses

Compared with baseline, the GH score was significantly higher at SVR24 (Table 2). Furthermore, the difference between baseline and each timepoint is shown in Fig. 1, and the differences in the GH scores at EOT and SVR24, the RE score at EOT, and the MH scores at EOT and SVR24 from the baseline values were significant (Fig. 1).

An assessment of the long-term effects of DAA treatment on HRQoL, that is, HRQoL at 1-year post-SVR24, was then conducted. At 1-year post-SVR24, only the GH score was significantly higher than the corresponding Japanese national standard value, whereas the PF, RP, SF, and RE scores and PCS were all significantly lower. The average BP, GH, VT, and MH scores and MCS at 1-year post-SVR24 were higher than the corresponding Japanese national standard values (Table 2); compared with the number of factors higher than the national standard value at baseline, two more factors were higher at this timepoint. However, the higher values in these categories did not equate to significant differences between baseline and 1-year post-SVR24 in the study cohort (Table 2, Fig. 1).

**Fig. 2:**
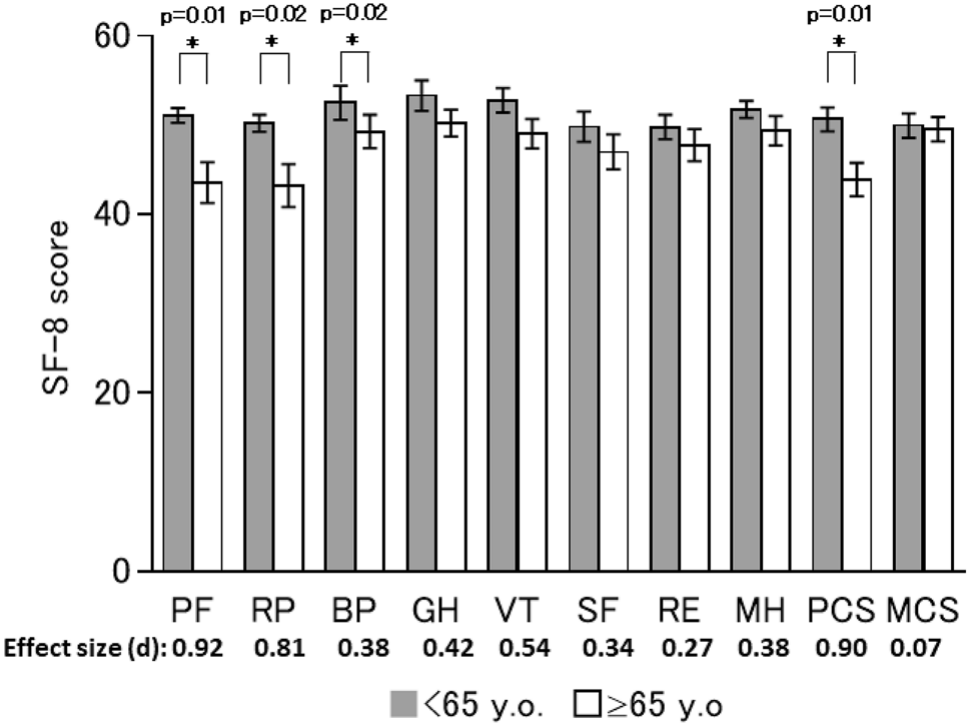
HRQoL score at 1-year post-SVR24 stratified by age. The mean SF-8 scores at 1-year post-SVR24 in participants aged < 65 years or ≥ 65 years. *p < 0.05 in a Student’s *t*-test. Error bars show the standard error

In addition, sub-analyses were performed to assess the potential effects of age, sex, liver status, and treatment regimen. Regarding age, participants were divided into two categories: ≥ 65 years and < 65 years old. There were significant differences in the PF (*p* = 0.01), RP (*p* = 0.20), and BP (*p* = 0.02) scores and in the PCS (*p* = 0.01) at 1-year post-SVR24 (Fig. 2) between patients aged ≥ 65 years and those aged < 65 years. VT was the only category to show significant differences in the scores between the two age groups (*p* = 0.04) from baseline to 1-year post-SVR24 (Table 3).

**Table 3:** HRQOL score at 1-year post-SVR24 stratified by factors (*n* = 42)

		PF	RP	BP	GH	VT	SF	RE	MH	PCS	MCS
Age	< 65 y.o. (*n* = 19)	− 0.03	− 1.05	− 0.08	2.38	2.45^§^	0.36	0.06	0.74	0.05	1.10
	≥ 65 y.o. (*n* = 23)	− 2.65	− 3.68	− 2.69	− 0.06	− 2.09^§^	− 0.90	− 1.24	− 0.88	− 3.09	− 0.06
	Effect size (d)	0.30	0.28	0.28	0.32	0.67	0.17	0.21	0.26	0.48	0.18
Sex	Male (*n* = 15)	− 1.15	− 3.53	− 0.95	3.34	2.23	− 1.38	− 2.62	− 0.20	− 0.74	− 0.34
	Female (*n* = 27)	− 1.64	− 1.91	− 1.82	− 0.23	− 1.29	0.26	0.44	− 0.12	− 2.19	0.91
	Effect size (d)	0.06	0.18	0.09	0.52	0.51	0.22	0.46	0.01	0.18	0.19
Liver status	CH (*n* = 30)	− 1.25	− 1.33	− 1.15	0.94	0.50	0.46	0.03	0.16	− 1.21	0.95
	LC (*n* = 12)	− 2.02	− 5.38	− 2.40	1.30	− 1.37	− 2.30	− 2.34	− 0.93	− 2.83	− 0.74
	Effect size (d)	0.07	0.35	0.13	0.05	0.26	0.35	0.33	0.14	0.16	0.22
Treatment regimen	LDV/SOF (*n* = 36)	− 0.97	− 2.18	− 1.58	0.99	− 0.81	− 0.38	− 0.79	− 0.91	− 1.19	− 0.31
	SOF/RBV (*n* = 6)	− 4.42	− 4.36	− 1.06	1.38	4.63	0.00	0.19	4.40	− 4.52	5.14
	Effect size (d)	0.43	0.21	0.05	0.39	0.87	−	0.15	1.00	0.30	0.83

Data are expressed as the mean of absolute differences between baseline and 1-year post-SVR24

*PF* physical functioning, *RP* role physical, *BP* bodily pain, *GH* general health, *VT* vitality, *SF* social functioning, *RE* role emotional, *MH* mental health, *PCS* physical component score, *MCS* mental component score, *CH* chronic hepatitis, *LC* liver cirrhosis, *LDV* ledipasvir, *SOF* sofosbuvir, *RBV* ribavirin

^§^*p* = 0.04 in a Student’s *t*-test

**Fig. 3:**
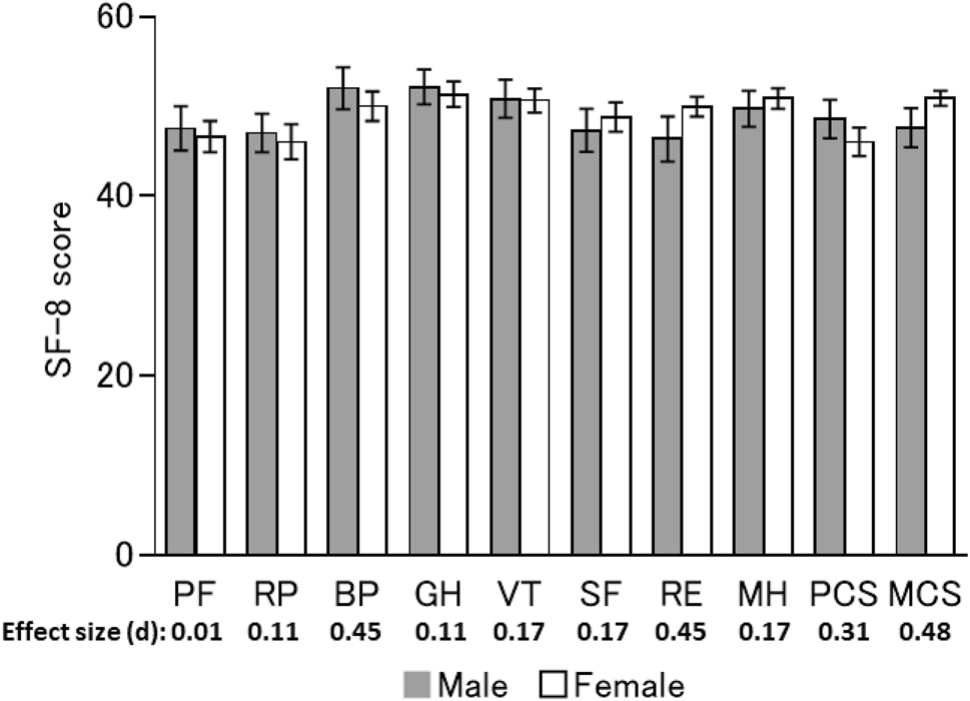
HRQoL score at 1-year post-SVR24 stratified by sex. The mean SF-8 scores at 1-year post-SVR24 in males or females. Error bars show the standard error

**Fig. 4:**
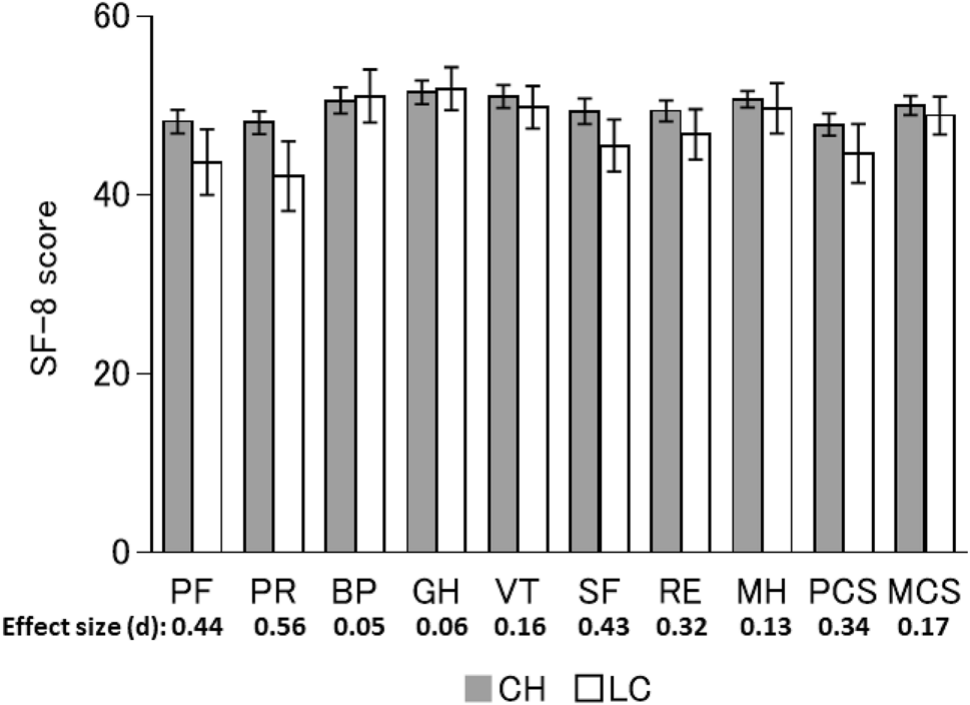
HRQoL score at 1-year post-SVR24 in CH and LC. The mean SF-8 score at 1-year post-SVR24 in participants with CH or LC. Error bars show the standard error

**Fig. 5:**
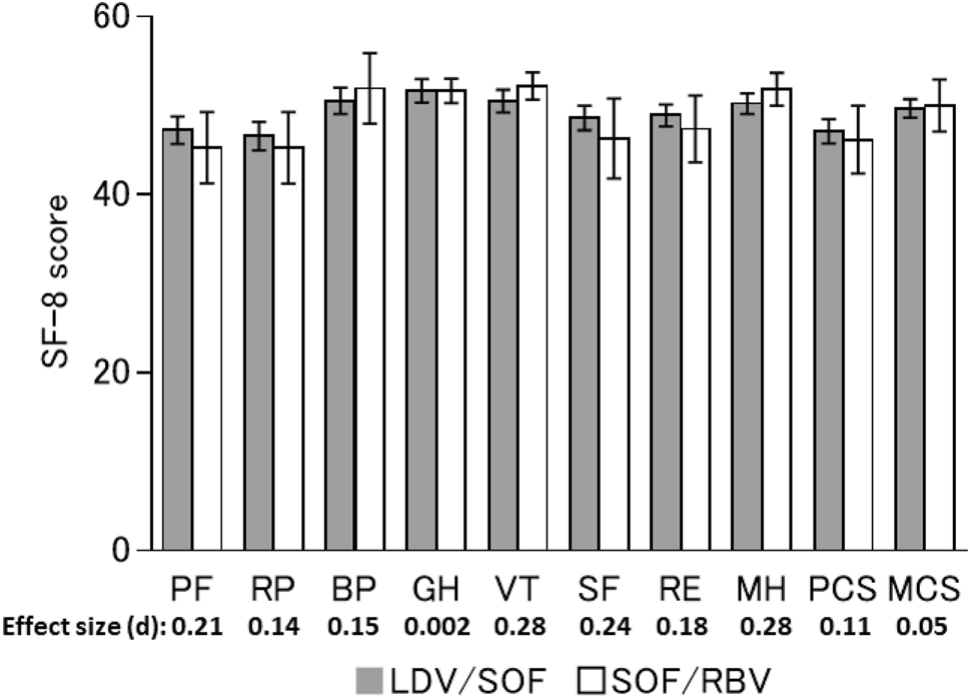
HRQoL score at 1-year post-SVR24 in patients stratified by treatment type. The mean SF-8 scores at 1-year post-SVR24 in participants who underwent IFN-free and RBV-free (LDV/SOF) or RBV-containing (SOF/RBV) treatment regimens. Error bars show the standard error

Regarding sex, liver status, and treatment regimen, there were no significant differences in any scores at 1-year post-SVR24 between males and females (Fig. 3), between LC and CH patients (Fig. 4), or between recipients of different treatment regimens (Fig. 5). Furthermore, there no significant differences between any of the baseline and 1-year post-SVR24 scores (Table 3) for these stratified groups.

A multivariable analysis was performed to detect factors (age, sex, liver status, and treatment regimen) with potential to be associated with influencing in the PCS and MCS at 1-year post-SVR24. Although none of these factors were found to be significantly associated with the MCS, age < 65 years old (odds ratio = 0.0335, 95% confidence interval: 0.00251–0.446, *p* = 0.0102) was identified as a significant factor associated with influencing the PCS (Table 4).

**Table 4:** Results of multivariable analyses of factors associated with influencing the PCS and MCS at 1-year post-SVR24

	PCS			MCS		
Factors	Odds ratio	95% CI	*p*-value	Odds ratio	95% CI	*p*-value
Age						
< 65 years old	1			1		
≥ 65 years old	0.0335	0.00251–0.446	0.0102	0.656	0.1050–4.08	0.651
Sex						
Female	1			1		
Male	0.2860	0.02600–3.140	0.3050	0.439	0.0760–2.54	0.358
Liver state						
CH	1			1		
LC	0.3110	0.05520–1.750	0.1850	0.572	0.1120–2.92	0.502
Treatment regimen						
LDV/SOF	1			1		
SOF/RBV	1.0300	0.14300–7.360	0.9800	0.244	0.0246–2.43	0.229

*PCS* physical component score, *MCS* mental component score, *CI* confidence interval, *CH* chronic hepatitis, *LC* liver cirrhosis, *LDV* ledipasvir, *SOF* sofosbuvir, *RBV* ribavirin

## Discussion

At baseline in this study, patients with chronic HCV infection had higher BP, VT, and MH scores compared with the Japanese national standard values. The baseline scores suggest that the participants of our study group were nearly pain free, with plenty of energy and little anxiety or mental stress. Although there is no clear evidence to support this possibility, we speculate that these characteristics could indicate that the original patients who were CH or compensatory LC had those characteristics or that the patients were positively influenced by the expected elimination of HCV by DAA treatment.

It has been reported that DAA treatment does not decrease HRQoL during treatment [8]. Other reports have shown that HRQoL was improved at SVR12–24 [9, 10]. In the present study, significant differences in the GH, RE, and MH scores between baseline and EOT were observed. Moreover, there were significant differences in GH and MH scores, but not in the PCS and MCS between baseline and SVR24. This significant elevation in the GH and MH scores between baseline and EOT or SVR24 was considered an effect of HCV elimination. Moreover, we conducted an assessment on the long-term effects of DAA treatment on HRQoL. The number of categories in which the scores were higher than the national standard values increased at 1-year post-SVR24 as compared with baseline, but there were no significant differences in any of the scores between baseline and 1-year post-SVR24. Thus, HRQoL slightly improved at 1-year post-SVR24 when compared with the national standard value.

Sub-analyses revealed no significant differences in any of the scores at 1-year post-SVR24 between the groups or between the baseline and 1-year post-SVR24 values in any category except for age.

Regarding aging, although there are reports of younger patients with hepatitis C [9–11, 15], many Japanese individuals with hepatitis C are elderly. In this study, subjects aged ≥ 65 years old accounted for 56% of subjects at baseline and 55% at 1-year post-SVR24. When investigating the scores at 1-year post-SVR24, we found that the PCS was significantly higher in the < 65-year-old group compared with older patients. VT was the only category to show a significant difference between the baseline and 1-year post-SVR24 values.

Regarding sex, higher baseline scores compared with the national standard values were identified in BP, VT, and MH for both male and female patients, but female patients also had a higher MCS. At 1-year post-SVR24, no significant differences from the national standard values were evident from the data, but categories with scores that trended higher than the national standard values were BP, GH, and VT in males and BP, GH, VT, RE, MH, and MCS in females.

Upon comparing patients with CH and those with LC, no significant differences in long-term HRQoL assessment were evident. This assessment may have been affected by the fact that decompensated liver cirrhosis was not included in this study.

Regarding treatment regimen, there have been several reports that RBV-containing regimens reduce HRQoL during treatment, but HRQoL increases after treatment [10, 11, 15]. This is likely a result of adverse events experienced during treatment. The lack of significant differences between these patient groups in the present long-term HRQoL assessment may indicate that the effects of adverse events are reduced at 1-year post-SVR24.

Notably, regarding age, sex, liver status, and treatment regimen, no significant differences were observed in the PCS or MCS between baseline and 1-year post-SVR24 for the pair of stratifications in each group. However, the PCS tended to decrease in the ≥ 65 years old, female, and LC groups. Conversely, the MCS tended to decrease in male patients. Therefore, patients in these groups should be observed carefully. Furthermore, only age < 65 years old was significantly associated with an improvement in the PCS at 1-year post-SVR24, whereas no significant factors were extracted for the MCS. Because a decreased PCS tends to be associated with advanced age, the PCS should be carefully monitored in patients over 65 years old.

This study has several limitations. First, it was single-center study. Second, there was a high attrition rate among the included cohort during the follow-up period. Consequently, although the required number of cases for appropriate evaluation of the long-term effect of DAA on HRQoL has been satisfied, the number of cases at 1-year post-SVR24 was small. However, the analyses other than the change in SF-8 score from baseline to 1-year post-SVR24 using collective group scoring can be performed in consecutive small cases. Third, we used an SF-8 to evaluate the HRQoL of patients. However, HRQoL is composed of comprehensive QoL (e.g., SF-8, SF-36, EQ-5D [16]) and disease-specific QoL (e.g., chronic liver disease questionnaire [17], cirrhosis-related symptom score [18], HepDisk [19]). Disease-specific QoL is highly responsive because the items focus on disease-specific symptoms. However, we needed to use comprehensive scales that could measure any unexpected negative efficacy. Furthermore, the SF-8 score can be compared with the Japanese national standard value of the SF-8. Ideally, we should have also considered assessing disease-specific QoL as well as comprehensive QoL.

In conclusion, upon long-term HRQoL assessment, the number of factors that were higher than the national standard value was increased at 1-year post-SVR24 than that at baseline, but there were no significant changes in any scores between the initiation of treatment and 1-year post-SVR24. However, a decreased PCS tended to be associated with advanced age, and so the PCS should be carefully monitored in patients aged over 65 years.

## Data Availability

Please contact author for data requests.
